# Environmentally triggered variability in the genetic variance–covariance of herbivory resistance of an exotic plant *Solidago altissima*

**DOI:** 10.1002/ece3.6130

**Published:** 2020-03-02

**Authors:** Yuzu Sakata, Shunsuke Utsumi, Timothy P. Craig, Joanne K. Itami, Mito Ikemoto, Takayuki Ohgushi

**Affiliations:** ^1^ Center for Ecological Research Kyoto University Otsu Japan; ^2^ Department of Biological Environment Akita Prefectural University Akita Japan; ^3^ Field Science Center for Northern Biosphere Hokkaido University Horokanai Japan; ^4^ Department of Biology University of Minnesota Duluth MN USA; ^5^ Department of Life and Environmental Sciences University of Tsukuba Tsukuba Japan

**Keywords:** biological invasion, G‐matrix, plant defense, plant‐insect interaction, reciprocal transplant experiment, *Solidago altissima*

## Abstract

The variability in the genetic variance–covariance (G‐matrix) in plant resistance and its role in the evolution of invasive plants have been long overlooked. We conducted an additional analysis of the data of a reciprocal transplant experiment with tall goldenrod, *Solidago altissima*, in multiple garden sites within its native range (USA) and introduced range (Japan). We explored the differences in G‐matrix of resistance to two types of foliar herbivores: (a) a lace bug that is native to the USA and recently introduced to Japan, (b) and other herbivorous insects in response to plant origins and environments. A negative genetic covariance was found between plant resistances to lace bugs and other herbivorous insects, in all combinations of garden locations and plant origins except for US plants planted in US gardens. The G‐matrix of the resistance indices did not differ between US and Japanese plants either in US or Japanese gardens, while it differed between US and Japanese gardens in both US and Japanese plants. Our results suggested that the G‐matrix of the plant resistance may have changed in response to novel environmental differences including herbivore communities and/or other biotic and abiotic factors in the introduced range. This may have revealed a hidden trade‐off between resistances, masked by the environmental factors in the origin range. These results suggest that the stability of the genetic covariance during invasion, and the environmentally triggered variability in the G‐matrices of plant resistance may help to protect the plant against multiple herbivore species without changing its genetic architecture and that this may lead to a rapid adaptation of resistance in exotic plants. Local environments of the plant also have a critical effect on plant resistance and should be considered in order to understand trait evolution in exotic plants.

## Graphical abstract

This article demonstrated that the novel herbivore environment in the introduced range can alter the genetic variance–covariance of resistance traits in an invasive plant can, while it remained stable during invasion. This suggests that the stability and variability of the genetic basis of resistance as an underlying mechanism for the rapid adaptation of resistance in invasive plants.

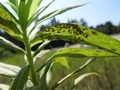

## 1 INTRODUCTION

Plants are usually attacked by multiple herbivores, and the herbivore communities differ geographically (e.g., Anstett, Naujokaitis‐Lewis, & Johnson, 2014; Craig, 2016; Strauss & Irwin, 2004). Therefore, different plant populations are subjected to different local selective pressures by herbivores. Plants either evolve resistance to specific herbivores through a pairwise (co)evolutionary arms race or respond simultaneously to multiple herbivores through diffuse (co)evolution (Agrawal, 2005; Berenbaum & Zangerl, 2006; Strauss, Sahli, & Conner, 2005). Diffuse (co)evolution occurs if the presence of a third species indirectly alters the magnitude and/or direction of natural selection or the response to selection in a pair of interacting species (Iwao & Rausher, 1997; Stinchcombe & Rausher, 2001; Strauss et al., 2005). In other words, regardless of how many species are involved, if (co)evolutionary interactions between the pair are not influenced by the presence of a third species, the interactions are considered pairwise rather than diffuse (Wise & Rausher 2013). One of the major factors that contributes to the trajectory of diffuse (co)evolution in plant resistances is the genetic correlations among plant resistances toward different herbivores. For example, a negative genetic correlation between two plant resistance traits can constrain evolutionary responses of the traits against two herbivores while each herbivore species can also impose directional selection toward higher resistance. Previous studies have found that plant resistances often have significant negative or positive genetic correlations (Franks, Wheeler, & Goodnight, 2012; Leimu & Koricheva, 2006; Stinchcombe & Rausher, 2001, Wise & Rausher 2013, Poelman & Kessler, 2016). The genetic covariance between plant resistances may potentially affect the evolution of plant resistance depending on the direction of selection (Conner, 2012).

These variances and covariances in plant resistance can be summarized in a genetic variance–covariance matrix (G‐matrix). This is a fundamental parameter in evolutionary quantitative genetics because G‐matrix constrains evolutionary responses of plants to natural selection (Lande, 1979). However, the extent of G‐matrix constraints on adaptation in plant resistance is still largely unknown. The breeder's equation in quantitative traits is defined as follows: ∆$\bar{z}$ = *Gβ*, where ∆$\bar{z}$ is the changes in the mean value of traits across one generation, *G* is the G‐matrix, and *β* is the vector of selection gradients (Lande, 1979; Lande & Arnold, 1983). Thus, phenotypic responses would vary owing to the G‐matrix even under equal selection gradients. Exploring population divergence patterns in G‐matrices can improve our understanding of the mechanisms underlying phenotypic divergence and how the response to selection might be constrained by genetic architecture during adaptive evolution (Eroukhmanoff, 2009; Schluter, 1996; Steppan, Phillips, & Houle, 2002). Several studies have found that the structure of the G‐matrix is stable (i.e., no changes in its direction and strength of variance and covariance) over ecological timescales of a few generations, while it is unstable in evolutionary timescales of hundreds of generations (Björklund, 1996; Cano, Laurila, Palo, & Merilä, 2004). On the other hand, recent studies have indicated that G‐matrix can change very rapidly over a few generations (Eroukhmanoff & Svensson, 2011; Phillips, Whitlock, & Fowler, 2001; Sgro & Blows, 2004; Uesugi, Connallon, Kessler, & Monro, 2017). In addition to the genetic changes in the G‐matrix, environmental conditions such as temperature and interacting species may also influence the structure of the G‐matrix because the environment affects gene expression (Bégin & Roff, 2001; Czesak & Fox, 2003; Wood & Brodie, 2015). Thus, environments are important in determining the correlations among resistances (Wood & Brodie, 2015). Understanding whether the G‐matrix in plant resistance is stable in different local populations and their environments is critical for making inferences about the evolution of plant resistance to herbivores. Reciprocal transplant experiments in which plant individuals from more than two populations are grown in their own environment and in the environments of the other populations, allow a clear separation of genetic and environmental effects on traits (Kueffer, Pyšek, & Richardson, 2013; Nuismer & Gandon, 2008). These experiments provide the ability to test whether G‐matrix varies among different populations or environments.

Plant invasions are excellent systems for studying how G‐matrix varies genetically or environmentally across local populations under different abiotic and biotic selection regimes (Eroukhmanoff & Svensson, 2011). Invasive plants may evolve rapidly in response to changes in biological interactions (Mitchell et al., 2006). The underlying mechanisms of this rapid evolution in resistance during invasion may be based on both the response to altered selection of a single resistance trait and changes in the G‐matrix of multiple resistance traits. Franks et al. (2012) compared secondary compounds in *Melaleuca quinquenervia* between its native range in Australia and invasive range in the USA and found that the genetic variances and covariances were reduced in the invasive range. They also found differences in the G‐matrices of plants between the invasive and native populations. The loss of genetic variation may be evidence of recent adaptation as well as founder effects (Franks et al., 2012). In addition, the reduction of negative genetic correlation may be a result of release from an evolutionary constraint affecting multiple resistance traits. This may potentially lead to the rapid evolution of plant resistance during invasion. Therefore, it is critical to explore the genetic and environmental variation in the G‐matrices between native and introduced plant populations in order to understand the rapid evolution of introduced genotypes in the invasive ranges.

**Figure 1:**
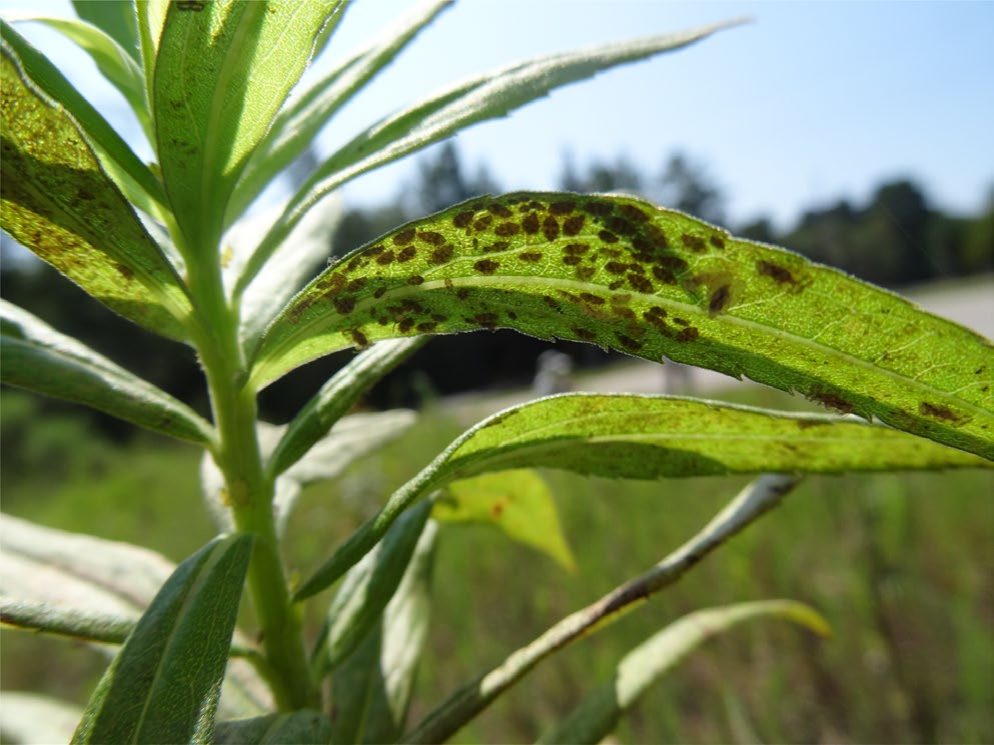
Lace bugs (*Corythucha marmorata*) feeding on *Solidago altissima* leaves

Tall goldenrod, *Solidago altissima*, is an herbaceous perennial native to old‐field habitats in North America. Several studies have found large genetic variability in goldenrod's resistance to insect herbivores (Craig, Itami, & Craig, 2007; Maddox & Root, 1987; Uesugi, Poelman, & Kessler, 2013; Utsumi, Ando, Craig, & Ohgushi, 2011). In Japan, *S. altissima* was introduced approximately 100 years ago and it has extensively invaded abandoned fields across the country (Shimizu, 2003). The lace bug, *Corythucha marmorata* (Hemiptera: Tingidae) (Figure 1), is one of the major herbivorous insects feeding on leaves of *S. altissima* in its native range of North America (Cappuccino & Root, 1992). It was introduced to Japan in 2000 and is now a dominant herbivorous insect in Japan. Although the specific resistant trait in *S. altissima* against lace bugs is unknown, secondary chemical compounds rather than physical traits are likely responsible for the resistance. This is because we did not find any relationships between physical traits (e.g., leaf trichome or leaf toughness) and resistance to lace bugs (Sakata, Yamasaki, & Ohgushi, 2016). In addition, Uesugi and Kessler (2016) found that Japanese *S. altissima* with low resistance to lace bugs showed lower production of leaf secondary metabolites such as diterpene acids, which may also affect resistance to other herbivorous insects. Diverse taxa of herbivorous insects were observed feeding on the plant in the USA, but very few taxa were observed in Japan (Sakata, Craig, Itami, Yamasaki, & Ohgushi, 2017). However, lace bug density was higher in Japan compared to the USA. Our previous study examining the relationship between plant resistances to lace bugs and other foliage feeding insects in multiple gardens in the USA and Japan showed an antagonistic relationship between them, which differed in strength among gardens (Sakata et al., 2018). These results suggest that the response of *S. altissima* to selection by lace bugs may differ between environments including differences in the herbivorous insect communities. However, it is not clear whether a negative genetic covariance actually exists and/or that degree differs among plant origins and environments. Thus, we hypothesized that a negative genetic covariance exists between resistance to lace bugs and other herbivorous insects and that its degree differs across native and introduced ranges and aimed to test this in this study. We conducted an additional analysis of the data of the resistances of *S. altissima* to herbivorous insects in a reciprocal transplant experiment with multiple replicates within the native and introduced ranges (Sakata et al., 2018), and specifically asked whether G‐matrices of the plant resistance differed between (a) origin populations of *S. altissima* in its native and introduced ranges, and (b) environments, which are reflected as the locations of the gardens, in its native and introduced ranges.

## 2 MATERIALS AND METHODS

### 2.1 Multiple reciprocal transplant experiment

From June to August 2013, we collected rhizome segments of *S. altissima* belonging to 10 genotypes from clumps at least 5 m apart from two populations in the USA (Minnesota, Kansas), and three populations in Japan (Saga, Shiga, Yamagata). Lace bugs were abundant on *S. altissima* in populations of Kansas, Saga, and Shiga, while they were absent or at low densities in populations of Minnesota and Yamagata (Sakata et al., 2017, 2016). The rhizome segments were planted in a greenhouse at the Center for Ecological Research, Kyoto University, Japan and at the Research and Field Studies Center, University of Minnesota Duluth (Table 1), followed by cultivation for two growing seasons to remove historical effects. In April 2015, the rhizomes were cut into 6 cm long segments with an average diameter of 5 mm; 25 ramets of each genotype were planted in pots and grown in the green house, and in June 2016, five ramets of approximately the same size from each genotype (250 plants in total) were planted in larger sized pots with potting soil and placed randomly in each of the five gardens (Table 1) (see Sakata et al., 2018 for detailed methods for cultivating the plants in the gardens).

**Table 1:** Location of the five gardens used in the reciprocal transplant experiment

State/Prefecture	Country	Latitude	Longitude
Minnesota	USA	N 46.86	W 92.03
Kansas	USA	N 39.22	W 96.61
Yamagata	Japan	N 38.69	E 139.82
Shiga	Japan	N 34.97	E 135.96
Saga	Japan	N 33.24	E 130.28

At the end of July, the number of leaves damaged by galls, mines, and chewing damage, excluding lace bug damage (which we term “other herbivore foliage damage”) were recorded for each ramet. Lace bug herbivory can be distinguished from other insect herbivory by their yellow feeding scars. The level of lace bug damage was assessed by assigning the damaged leaves to four levels: (a) no damage, (b) <33% damage, (c) 33%–66% damage, and (d) >66% damage of total leaf area. Subsequently, we counted the number of leaves in each damage level, added the values of all four levels (which we term “lace bug damage”), and divided that with the total number of leaves to calculate the average damage value per leaf for each plant. For other herbivore foliage damage, we simply divided other herbivore foliage damage by the total number of leaves. We also counted the total number of leaves on all plants in each garden. For further analyses, we used these two values as resistance indices for herbivorous insects.

### 2.2 Computation and comparisons of G‐matrix in plant resistance

Genetic variances and covariances of the two resistance indices (i.e., lace bug and other herbivore herbivores) for (a) US plants in US gardens, (b) US plants in Japanese gardens, (c) Japanese plants in US gardens, and (d) Japanese plants in Japanese gardens were estimated. For this purpose, we applied a multivariate, random effects model in a Bayesian‐MCMC framework (animal models implemented in the MCMCglmm; Hadfield, 2010). For each G‐matrix, (a) 20 genotypes with ten replicates, (b) 20 genotypes with 15 replicates, (c) 30 genotypes with ten replicates, (d) 30 genotypes with 15 replicates were used with population identity being included as a random effect. We acknowledge that using the Bayesian approach with pooling may not yield a strict estimate of G‐matrices for populations because the populations within countries are geographically apart, and genotypes from each population may not be statistically independent of one another if there is within population correlation of genotypic variation. However, this approach does provide informative estimates for large‐scale trends such as changes in G‐matrices through invasion from the base native range to introduced range across continents (Careau, Wolak, Carter, & Garland, 2015). To estimate G‐matrices between traits, we implemented a bivariate version of the animal model (Wilson et al., 2009) described by Eq. 1 in MCMCglmm (Hadfield, 2010), which included lace bug damage and other herbivore foliage damage as response variables assuming a Poisson distribution, and number of leaves (offset term), population origin and garden location (random factor) as independent variables. We used noninformative inverse Wishart distribution priors (Hadfield, 2010). For the offset term, priors with a diffuse normal distribution centered around zero and with a very large variance (10^8^) were used. All Markov chain Monte Carlo (MCMC) analyses were run for 65,000 steps with 15,000 discarded as burn‐in, and chains were thinned by selection every 50 steps, yielding a total of 1,000 data points for each analysis. We checked the plots of the traces and posterior distributions and calculated the autocorrelation between samples, and we confirmed that all models properly converged. In this model, the phenotypic matrix P contains the phenotypic variances and covariances between traits. This matrix can be decomposed into an additive genetic matrix (G) and a residual (or environmental) matrix (R), so that P = G + R. In this model, we obtained a posterior distribution of 1,000 matrices that summarizes the uncertainty in the estimation of respective G‐matrices. In accounting for fixed effects and calculating G‐matrices on the observed scale upon which traits are measured for non‐Gaussian trait distribution in the GLMM (i.e., Poisson with log link function), it is necessary to obtain accurate G‐matrices (de Villemereuil, Schielzeth, Nakagawa, & Morrissey, 2016). Thus, we estimated accurate G‐matrix parameters on the observed scale by looping the “QGmvparams” function in the “QGglmm” package in R (de Villemereuil et al., 2016). Because calculating G‐matrices on the observed scale takes a long time (i.e., 48 hr for ten MCMC samples), we reran MCMC analyses for 65,000 steps with 15,000 discarded as burn‐in, and chains were thinned by selection every 5,000 steps, yielding a total of 10 data points to estimate the distribution of G‐matrices on the observed scale. Note that similar results were obtained with the posterior distribution of 1,000 matrices. As a result of estimating the G‐matrices based on clonal replicates, they are broad‐sense genetic parameters that include additive and nonadditive genetic effects plus shared environmental effects (Lynch & Walsh, 1998). Although using clonal replicates overestimates the genetic variances, studies have found that the breeding design does not affect the magnitude of genetic correlations between resistances (Leimu & Koricheva, 2006).

To test whether the structure of G‐matrix of *S. altissima* has been genetically and/or environmentally altered following introduction to Japan and to explore potential constraints on trait evolution, we compared the G‐matrices as follows: (a) US plants in US versus. Japanese gardens, (b) US plants versus. Japanese plants in US gardens, (c) Japanese plants in US versus. Japanese gardens, and (d) US versus. Japanese plants in Japanese gardens. We used a Bayesian statistical framework of Krzanowski subspace method for comparing the G‐matrices. This approach can provide a robust comparison withstanding the uncertainty of the genetic parameters that are estimated and examines differences in G‐matrices with respect to their orientation (Aguirre, Hine, McGuigan, & Blows, 2014). The Krzanowski subspace method examines if the majority of genetic variance in trait space is shared across groups (Krzanowski, 1979). It measures the similarity of the space spanned by the first several eigenvectors of the matrices being compared. We calculated Krzanowski's similarity index (H), which is a matrix that contains the eigenvectors of each G‐matrix. The eigenvalues of H are bounded by the number of matrices being compared (i.e., the maximum number is two here). For each eigenvectors of H, eigenvalues can be calculated (see the supporting information in Aguirre et al. (2014) for detailed methods). We used the first eigenvector, which is less than half of the number of dimensions (i.e., two: lace bug and other damage) as recommended by Krzanowski (2000). To test if the matrices share the same subspace, we followed Aguirre et al. (2014) and constructed a randomized set of matrices (H). We assumed that all matrices were sampled from the same group and combined the 10 G‐matrices created by the MCMC analyses for each group and randomly assigned individuals to one of the groups and constructed G‐matrices from the vectors of breeding values (genotype). If the randomized and observed eigenvalues of H were the same, we concluded that the matrices share the same subspace. All statistical analyses were conducted in R 3.3.2 (R Development Core Team, 2016).

## 3 RESULTS

### 3.1 G‐matrices among plant resistances to foliar herbivores

We obtained four G‐matrices in terms of all combinations of plant origins and gardens (Table 2). All covariances between resistance indices were significantly different from zero. There were no large differences between the MCMC analyses with 1,000 samples and 10 samples (Table 2 vs. Table S1). A negative genetic covariance was detected between plant resistances to lace bugs and other herbivorous insects in all G‐matrices except for that of the US plants in US gardens, where a positive genetic covariance was detected (Table 2). The genetic variances of resistance to lace bugs were larger in Japanese gardens than in US gardens, whereas the genetic variances of resistance to other herbivorous insects were larger in US gardens than in Japanese gardens (Table 2).

**Table 2:** Observed scale G‐matrices for resistance indices of *S. altissima* obtained from the 1,000 samples of posterior distribution of the MCMC analyses of (a) US plants in US gardens, (b) US plants in Japanese gardens, (c) Japanese plants in US gardens, and (d) Japanese plants in Japanese gardens

	Lace bug	Other		Lace bug	Other
(a) US plants in US gardens			(b) US plants in Japanese gardens		
Lace bug	*2.87 (0.47, 13.21)*	0.01 (0.003, 0.09)		*1.17e^4^ (1.51e^3^, 2.96e^4^)*	−0.69 (−2.69, −0.13)
Other		*3.26 (0.43, 12.25)*			*0.09 (0.01, 0.23)*
(c) Japanese plants in US gardens			(d) Japanese plants in Japanese gardens		
Lace bug	*0.028 (0.001, 0.19)*	−0.01 (−0.07, −4.0e^−4^)		*61.67 (10.83, 184.3)*	−0.07 (−0.32, −0.02)
Other		*5.50 (0.25, 34.59)*			*0.21 (0.034, 0.59)*

Note

Genetic variances are in italics. All genetic variances and covariances are statistically significant at *p* < .05. Other: other foliage feeding herbivores. Values in parenthesis indicate 95% highest posterior density intervals.

**Figure 2:**
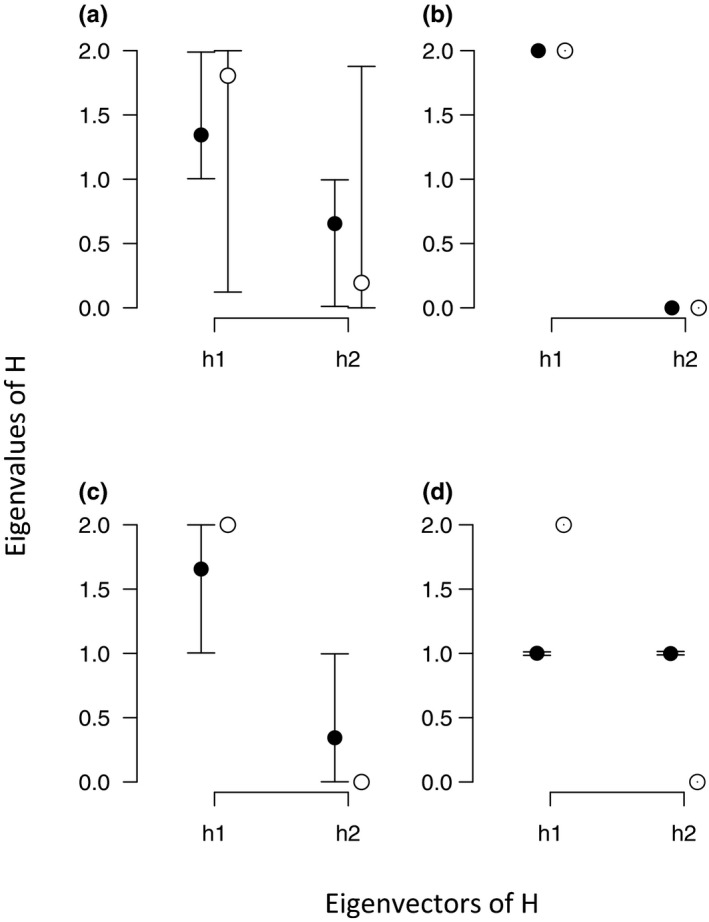
Posterior median (±95% highest probability density (HPD) intervals) eigenvalues (h1 and h2) of the summary matrix H, which contains the first eigen vectors of the G, obtained from the observed and randomized matrices of Krzanowski shared subspace observed: (a) US plants in US garden versus Japanese plants in US gardens, (b) US plants in Japanese gardens versus Japanese plants in Japanese gardens, (c) US plants in US gardens versus US plants in Japanese gardens, (d) Japanese plants in Japanese gardens versus Japanese plants in US gardens. Closed and open circles indicate observed matrices and randomized matrices, respectively. The 95% HPDinterval are (a) (h1) observed: 1.00–1.99, randomized: 0.087–2.00; (b) (h1) observed: 1.99–2.00, randomized: 1.99–2.00; (h2) observed: 4.71 × e^−7^–4.59 × e^−6^, randomized: 2.11 × e^−7^–5.48 × e^−6^; (c) (h1) observed: 1.00–1.9991; randomized: 1.9999–2.00; (h2) observed: 9.01 × e^−4^–0.99, randomized: 2.55 × e^−8^–8.60 × e^−6^; (d) (h1) observed: 0.98–1.01, randomized: 1.99–1.99; (h2) observed: 0.99–1.02, randomized: 4.74 × e^−7^–1.29 × e^−4^

According to the Krzanowski subspace method, the 95% HPD intervals for the eigenvectors of H overlapped between observed and randomized values for either the comparison of US plants in US gardens and Japanese plants in US gardens (Figure 2a), or the comparison of US plants in Japanese gardens and Japanese plants in Japanese gardens (Figure 2b). This indicates a shared subspace and stable set of eigenvectors between different plants origins. However, the 95% HPD intervals or the eigenvectors of H did not overlap between observed and randomized values when comparing US plants in US gardens and US plants in Japanese gardens (Figure 2c, 95% HPD interval (h1) observed: 1.00–1.9991, randomized: 1.9999–2.00; (h2) observed: 9.01 × e^‐4^–0.99, randomized: 2.55 × e^‐8^–8.60 × e^‐6^), and for the comparison of Japanese plants in US gardens and Japanese plants in Japanese gardens (Figure 2d). This indicates that the subspace of eigenvectors is diverged between the gardens.

## 4 DISCUSSION

The results showed that a negative genetic covariance was detected between lace bugs and other herbivorous insects, and the G‐matrix of the resistance indices did not differ between US and Japanese plants either in US or Japanese gardens, while it differed between US and Japanese gardens in both US plants and Japanese plants. This suggests that the G‐matrix may be stable during an invasion and variable among environments, which could be important mechanisms allowing for the rapid adaptation in invasive plants.

### 4.1 Negative genetic covariance between plant resistances to different herbivorous insects

A negative genetic covariance was found between resistances to lace bugs and other herbivorous insects in all combinations of plant origins and gardens except for US plants in US gardens. Because *S. altissima* shared a long evolutionary history with various herbivorous insects in its origin range including lace bugs, different specialized plant defensive traits may have coevolved with lace bugs, and other herbivorous insects. Thus, trade‐offs in investing in these traits could result in negative genetic covariance between specific resistance to lace bugs and other herbivorous insects. Note that as we categorized foliage feeding insects other than lace bugs in one category, we cannot eliminate the possibility that some foliage feeding insects share the same defensive traits with lace bugs leading to the absence of trade‐off with resistance to lace bugs. The large variance in the genetic covariance detected in our study may be due to this categorization. However, we note that even using this categorization of herbivores the negative genetic covariance was detected. Alternatively, the negative genetic covariance between herbivores may be due to behavioral avoidance of herbivory caused by other species (Ando, Utsumi, & Ohgushi, 2017; Halitschke, Hamilton, & Kessler, 2011; Poelman & Kessler, 2016). Note that even when lace bug damage was most prominent in Japanese gardens, undamaged leaves were unlikely to be scarce for other herbivores.

The small negative genetic covariance between the resistances to lace bugs and other herbivorous insects in US gardens could be explained by combined effects of environmental factors and evolutionary backgrounds. First, *S. altissima* in the USA is imposed by a similar level of herbivory by these two types of insects as reflected in our gardens in the USA (Sakata et al., 2018). Second, the long‐term directional selection acting to increase both resistances in the USA with the presence of negative covariance may have maintained both resistance levels at the intermediate levels. These two effects may have led to the covariance between resistances being close to zero in the US gardens. The reason why a positive genetic covariance was detected in US plants in US garden is unclear; however, since there were no differences in the G‐matrices between plant origins within the US gardens it is unlikely that this positive value has ecological significance. On the other hand, in the Japanese gardens with high abundance of lace bugs, the difference in the resistance as damage rate in the US plants may have been magnified, which allowed us to detect the negative covariance. Contrarily, in the case of Japanese plants, they have recently received a directional selection only toward the increase in lace bug resistance following the recent lace bug invasion. As a result, a negative genetic covariance might be realized along the antagonistic relationship of resistances in gardens of both countries.

An alternative explanation for the difference in covariance of resistance on US plants in US and Japanese gardens is that difference in the lace bug phenology between locations may cause difference in plant quality caused by other insect herbivory. Helmberger, Craig, and Itami (2016) reported that lace bugs perform better on drought‐stressed *S. altissima* due to mobilization of structural nitrogen increasing the nutritional quality of stressed tissues. In addition to drought stress, stress from early herbivory may positively affect lace bug performance, as early herbivory has been reported to increase nitrogen and increase herbivory by later herbivores (Danell & Huss‐Danell, 1985). Because lace bugs emerge later in the season in the USA compared to Japan (Y.S. personal observation), there may be a positive effect of stress due to previous damage by other herbivores on the lace bugs in the USA.

### 4.2 Stability of the G‐matrix during invasion

Interestingly, although the US plants in US gardens had a significant positive covariance and the others had a significant negative covariance, the overall G‐matrix of the resistance indices did not differ between US and Japanese plants either in US or Japanese gardens as indicated by the shared subspace and a stable set of eigenvectors of the G‐matrices. This may be because (a) the genetic variances were similar between the US and Japanese plants in each garden, (b) the covariances were relatively smaller than variances, and (c) covariances were close to zero in US gardens. Roff and Mousseau (1999) suggested that different cricket populations, which have different traits under clinal selection that show genetic covariances, displayed the unchanging G‐matrices because of the proportional change in both genetic covariances and variances. Although the genetic variance in lace bug resistance tends to be larger in US plants, the slightly larger genetic covariance may have led to the stability in the overall genetic architecture in resistances to lace bugs and other herbivorous insects between native and introduced plants in each of the US and Japanese gardens. Studies that detected rapid changes in the G‐matrix of plant secondary metabolites used plants that were either free from herbivores as a result of being introduced to a new range (Franks et al., 2012), or due to insect suppression (Uesugi et al., 2017). Uesugi et al. (2017) indicated that an artificially herbivore‐free habitat altered the orientation (i.e., direction and strength of the variance and covariance) of the G‐matrix of defensive traits in *S. altissima* and revealed a negative genetic covariance between defense‐ and competition‐related metabolites. On the other hand, since *S. altissima* populations in Japan were heavily attacked by the invasive lace bug, with which they had experienced a long evolutionary history in the native range, the evolution of resistance to the lace bugs due to reassociation may not have led to the change in the G‐matrix of the resistance traits. A comparison of our result with the above studies showing rapid changes in the G‐matrices suggests that the negative genetic covariance between resistances may not be altered unless herbivore‐free environment persists over several generations of plants and that condition is ongoing. Alternatively, the G‐matrix of the amount of each chemical compound such as the secondary metabolites may be more labile than that of the overall resistance level as a function of a vast array of many compounds. This could be attributed to overall resistance being comprised of multi‐layered components of traits such as the wide variety of chemical compounds and/or physical leaf defense.

Another factor that might have led to the similar G‐matrices for US and Japanese populations is continuous migration between populations in the two countries that may homogenize the genetic variation between ranges (Guillaume & Whitlock, 2007). However, the divergence of US and Japanese *S. altissima* populations was revealed by a neutral molecular genetic analysis, and the Japanese populations used in this study were genetically similar and likely shared a common origin from a single or a small number of US populations (Sakata, Itami, Isagi, & Ohgushi, 2015). It is, therefore, unlikely that migration between populations caused the similar G‐matrices in the USA and Japan.

### 4.3 Environmentally triggered variability in the G‐matrix between native and introduced ranges

Although the structure of G‐matrix did not differ between US and Japanese plants, it differed between US and Japanese gardens in both US plants and Japanese plants (i.e., as the subspace and a stable set of eigenvectors of the G‐matrices were not shared between gardens). This indicates that environmental differences influence the magnitude and the sign of G‐matrix. Together with our former study (Sakata et al., 2018), the genetic variances of both lace bugs and other herbivore resistances were larger in gardens with higher levels of insect damage. In fact, a higher genetic variance of resistance to lace bugs was found in Japanese gardens, and a higher genetic variance of resistance to other herbivorous insects was found in US gardens. This could be the primary mechanism producing the difference in the G‐matrix between US and Japanese gardens. Although the genetic covariance between lace bugs and other herbivorous insects was relatively larger in the Japanese gardens compared to the US gardens, the low damage rate by other herbivorous insects in the introduced range compared to the native range (Sakata et al., 2018) may have led to the release of constraints caused by the genetic covariance in response to selection imposed by the lace bugs. Therefore, contrary to our hypothesis, the difference in degree of genetic covariance may not cause either a large constraint or an augmentation to the response to selection as the genetic covariance between lace bugs and other herbivorous insects was small (close to zero) in all combinations of plant origins and gardens. In sum, changes in genes regulating resistance are not likely to be the mechanism responsible for the changes in the genetic correlation between environments. Rather, the degree of the gene expression of resistance may differ among populations with different insect communities. Many studies have reported an induction of resistance including phytohormonal crosstalk in response to an attack by multiple herbivore species (Bonaventure, Doorn, & Baldwin, 2011; Pieterse, Does, Zamioudis, Leon‐Reyes, & Wees, 2012; Stam et al., 2014). Our results are consistent with the notion that environmental variation is important for structuring trait correlations (Wood & Brodie, 2015).

## 5 CONCLUSIONS

The main reason why the G‐matrices differed between environments but it did not differ between plant origins may be that the genetic variance of lace bugs became large in Japanese gardens due to extremely high lace bug density, which can cause increase in gene expression and/or induction for lace bug resistance. Strauss et al. (2005) argued that changes in community composition can alter the G‐matrix of a trait under selection by one interactor in the context of diffuse evolution. Unexpectedly, our results suggest that the genetic covariance can be stable during invasion and may not impose a large constraint on the evolution of defense in invasive plants. This is likely because this stability in the trade‐off between plant defenses is favored in highly heterogeneous herbivory environments. Trade‐offs may be an underlying adaptive mechanism that evolved under spatiotemporal variation in the community structure of herbivores. In addition, the environmentally triggered variability in G‐matrices of plant resistance may promote plastic adaptation that increases resistance to specific herbivores in invasive plants. Although rapid evolution in invasive plants has been reported in many species (Mitchell et al., 2006), the mechanisms producing it is still poorly understood. These two characteristics of the G‐matrices that we found may be key mechanisms in invasive plants that allow them to quickly adapt to the new range. In this study, we compared the G‐matrices between native and introduced ranges, based on data from multiple gardens within each range that cover a wide range of different environments. This has enabled us to determine that novel environmental factors including the herbivore communities and/or other biotic and abiotic factors in introduced ranges can alter the G‐matrices of plant resistances. Moreover, this allowed us to detect a hidden trade‐off between the two resistances, which may have been masked by the environmental factors such as the composition of insect community in the origin range. In future, the G‐matrix of each population should be measured to more accurately understand the stability and variability of G‐matrices among different environments. The local environment, including the herbivore community, has a critical effect on plant resistance, and it should be considered in order to understand the trait evolution of invasive plants.

## CONFLICT OF INTEREST

None declared.

## AUTHOR CONTRIBUTIONS

TPC and YS designed and planned the experiment. YS, MI, TPC, and JKI conducted the field experiment. YS and SU analyzed the data. All authors contributed to writing and editing the manuscript.

## Data Availability

All data used in this manuscript are deposited to Dryad https://doi.org/10.5061/dryad.ht76hdrbt.
