# Supplementary material for: Environmentally triggered variability in the genetic variance–covariance of herbivory resistance of an exotic plant Solidago altissima
Source: Ecol Evol. 2020 Mar 2;10(6):3103–11. doi: 10.1002/ece3.6130 (PMC7083677; doi:10.1002/ece3.6130)
Supplement: Supplementary file 1 [file ECE3-10-3103-s001.docx]

Table S1. Observed scale genetic variance and covariance (G) for resistance indices of *S. altissima* obtained from the 10 samples of posterior distribution of the MCMC analyses of (a) USA plants in USA gardens. (b) USA plants in Japanese gardens, (c) Japanese plants in USA gardens, and (d) Japanese plants in Japanese gardens.

|  | | Lace bug | | Other |  | | Lace bug | Other |
| --- | --- | --- | --- | --- | --- | --- | --- | --- |
| (a) USA plants in USA gardens | | | | | (b) USA plants in Japanese gardens | | | |
| Lace bug | *4.26*  *(0.18, 20.78)* | | 0.31  (0.001, 2.40) | |  | | *1.23 e^4^*  *(1.91e^3^, 1.12e^4^)* | -1.19  (-3.93, -0.10) |
| Other |  | | *1.52*  *(0.60, 2.76)* | |  | |  | *0.09*  *(0.14, 0.49)* |
| (c) Japanese plants in USA gardens | | | | | (d) Japanese plants in Japanese gardens | | | |
| Lace bug | *0.09*  *(0.001, 0.44)* | | -0.02  (-0.19, -2.31e^-5^) | | |  | *1.46 e^3^*  *(9.88e^e^, 2.32e^3^)* | -1.10  (-4.60, -0.80) |
| Other |  | | *9.79*  *(8.58, 19.45)* | | |  |  | *0.13*  *(0.06, 0.23)* |

Note: Genetic variances are in italics. All genetic variances and covariances are statistically significant at P < 0.05. Other: other-herbivorous insects. Values in parenthesis indicate 95% HPD intervals.
